# Supplementary material for: Changes to Adult Dog Social Behaviour during and after COVID-19 Lockdowns in England: A Qualitative Analysis of Owner Perception
Source: Animals (Basel). 2022 Jun 29;12(13):1682. doi: 10.3390/ani12131682 (PMC9264766; doi:10.3390/ani12131682)
Supplement: Supplementary file 1 [file animals-12-01682-s001.zip › animals-1747145-supplementary.pdf]

Table S1. Primary and sub-themes, perceived outcomes and additional quotes concerning changes in adult dog behaviour as a result of COVID19 lockdowns in England identified through semi-structured interviews with participants (n=15).

Major Theme	Sub-Themes	Perceived Outcomes and Participants Reporting (n)	Quotes
Changes in environment	External changes	Increased walking frequency (4)	*"Even in the first part of the lockdown, he got walks every day. Long walks as well, rather than, I've got half an hour in between meetings, I'll take him out for a quick walk, not a long walk. So he was loving it all the way through lockdown because he had someone at home looking after him." - (R10)*
		Busier walking routes (5)	*"There's one particular location I will go to where suddenly in March 2020 it's almost like the whole of [city] suddenly discovered this place and it went from being somewhere which was really not very busy to somewhere which was always really busy." - (R9)*<br><br>*"Walks were busier, definitely. Well, it was busier where we are anyway. I think that's because people that were normally working were at home." - (R12)*<br><br>*"I would go on walks in the countryside, and I could bump into groups of people walking. And usually, that never happens around here, normally it's the occasional dog walker, and a bike." - (R15)*
		Quieter walking routes (3)	*"In the initial part of lockdown, it was much quieter. And I think that was mainly because people were unsure, didn't want to go out and wasn't sure what they were allowed to do." - (R10)*<br><br>*"Walks were quieter than before lockdown. You didn't see as many people where we used to." - (R1)*
	At home changes	Remote working (10)	*"Yeah, I used to work in the office probably three days a week. My wife does stay at home, but she would need to nip out every now and again. So it was at that point that she'd be on her own. Now, I'm very rarely in the office. You know, kind of been in three times I think in the last 18 months." - (R14)*<br><br>*"Before COVID I worked from home probably two days a week. And it changed week on week so there was never a formal [amount of time] that she was always on her own and then when the pandemic hit, I worked from home all the time, and so did my wife. So we were both at home all the time." - (R5)*<br><br>*"Before lockdown, I was at home and in the office about half and half. My husband does shifts. He's away when he's working but it's either mornings, afternoons or overnight. It's a mismatch of getting in and out. I started*

			working from home, my husband works on the railway, so he was still doing his shifts." - (R11)<br><br>*"Yeah, I used to work in the office probably three days a week. My wife does stay at home, but she would need to nip out every now and again. So it was at that point that she'd be on her own. Now, I'm very rarely in the office. I've been in three times I think in the last 18 months." - (R14)*
		Increased owner presence (8)	*"I used to work in the office four days a week and one day at home. Pre-COVID my wife used to work only half days. So he was only at home on his own for about three and a half hours on those days that I wasn't here. So he was loving it all the way through lockdown because he had someone at home looking after him" - (R10)*<br><br>*"Yes, I think he got a lot more company then. He just had companionship all the time for 24 hours a day, so I think he was pretty in his element with lots of fuss and attention." - (R3)*
Changes in dog-dog behaviour	Increased reactivity	Dog attack (2)	*"[My dog] had an incident where we were on the field and a dog ran up to her and she panicked and actually ran home from across this main road, and we were screaming." - (R1)*<br><br>*"One of the dogs [we passed on our walk] for whatever reason just took a dislike to [my dog]. My son had to literally prise this other dog's jaws off [my dog]. This woman was ever so apologetic. It was her dog that started it, but my dog retaliated." - (R4)*
		Increased fear (5)	*"His reactivity got worse, because he had quite a few bad encounters with off lead dogs, so he tended to get very worried by other dogs especially when they start getting giddy and excited." - (R15)*<br><br>*"He knows which dogs he does not like, not that they've ever done anything to him, but I think that is something that has changed a bit." - (R3)*
		Increased aggression (2)	*"Before lockdown, we'd be able to walk anywhere, and he would always be so happy to see dogs. Now when we take him around the block, there's a couple that he will bark at in a nasty way. And he never used to be like that." - (R13)*<br><br>*"Since lockdown he has snapped at other dogs." - (R3)*
	Decreased reactivity	Spent more time in the company of dogs (2)	*"She seemed to want to play with more dogs than she would have done previously." - (R5)*

			"She coped really well [with the new puppy]. It's the usual thing with the overexcited puppy jumping on her, but they established an order and there was no aggression. She welcomed [the puppy] in the house." - (R6)
		Less excitement toward dogs (1)	*"I know pre-lockdown they'd be overexcited about seeing another dog and definitely have to go over and say hello. And if the dog was on a lead, we'd be constantly putting them back on the lead, so they don't run over. Whereas now they do tend to stick with us more." - (R11)*
Changes in dog-human behaviour	Fear-related changes	Increased anxiety (4)	*"If she doesn't know someone, she stands off until I've spoken to them or occasionally, she's barked. It was an 'I'm not too sure' bark. And that's only happened since lockdown." - (R8)*<br><br>*"He hates the postman now and I don't know if that's just because he was one of the only people coming to our house. His hackles go up when the postman is there, which is weird because he's the nicest friendliest dog ever." - (R2)*
		Increased reactivity (3)	*"I think barking has got worse to be fair, at everyone." - (R2)*<br><br>*"I definitely noticed that she would bark a lot more at people walking past the house because there would be a lot more people walking past the house than there used to be, as they were trying to go onto the fields and onto their walk." - (R5)*<br><br>*"He's gone from barking when people are on the drive, to doing it when people are walking by our house. It could just be someone taking the kids to school and he's barking at them." - (R2)*
		Decreased reactivity (3)	*"Guests coming to the house was slightly better." - (R9)*<br><br>*"She's perhaps a little better behaved when we're opening the door. If you tell her to stay she quietens down and stays and doesn't always dart for the door to get out. Possibly because you get a lot more deliveries and food delivered." - (R7)*
		Relinquishment (1)	*"[My dog] wasn't constantly barking because it's a human [baby], it was more 'Can I play with this'. So that's the reason why we've ended up having to rehome her." - (R5)*

	Separation-related changes	Increased separation distress (1)	*"My wife's never left the house through lockdown and now when she does, [my dog is] a little bit more anxious, I've noticed that." - (R6)*
		Decreased separation distress (1)	*"If we do tend to leave the house now, there's no barking." - (R14)*
	Other changes	Increased excitement (4)	*"Now [when visitors arrive] it's completely over the top because she jumps into people's arms." - (R4)*<br><br>*"When people started coming back to the house, they were really gone. They've gone over excited and jumping at people. They're not used to people coming." - (R1)*
		Increased attention-seeking (4)	*"He did want attention more." - (R2)*<br><br>*"He'll come and sit in the office when I'm in the office. If I'm sat on the sofa or somewhere he can sit on me or next to me, he would do that. If I moved from room to room, he would follow." - (R3)*
		Increased calmness (3)	*"He has generally been more chilled out." - (R2)*
		Companionship (6)	*"He just had companionship all the time for 24 hours a day, so I think he was pretty in his element with lots of fuss and attention." - (R3)*
Changes in non-social behaviour	Training and behavioural intervention	Training regression (4)	*"He regressed in everything during lockdown, but It's pretty easy to get it back now we know what to do." - (R2)*<br><br>*"She used to pull shocking on the lead, we got her back on track and she stopped, but it's started again." - (R1)*
		Involvement of canine behaviourist (2)	*"We actually paid for a dog behaviour therapist to come around and give us some tips about how to get people in the house." - (R5)*<br><br>*"I've had to have a behaviourist out because of the anxiety issues." - (R1)*
	Fear-related changes	Fear of motorbikes (1)	*"He is terrible with motorbikes. They were trying to go on the trails where I would expect them every now and again, but it was to the point where I had to avoid that trail because they're coming here a lot." - (R15)*

		Fear of car journeys (5)	*"She was quite nervous in the car. And would sit a bit shaky, squealy because previously before lockdown she really only went in the car when we were going on a journey because I used to walk her locally." - (R8)*<br><br>*"She's not very good in the car. She's sick in the car. It has probably got worse because obviously, she's not in the car as much. In previous years she's not sick every time. But, she's been sick every time since." - (R7)*
Future concerns	Separation-related problems	Holidays (1)	*"It does worry me a bit because next year we're thinking we want to go on a foreign holiday. But then I'm worried because obviously [my dog is] used to us being [with her]." - (R4)*
		Return to conventional work (5)	*"You don't realise when you're at home with them all the time then suddenly you're not, what difference it makes." - (R1)*<br><br>*"I'd worry about going back to work full time. And if my husband didn't do shifts, about leaving them on their own for a full day. I think they would maybe struggle with that now because I can't think of the last time that happened." - (R11)*
